# 4,5-Dibromo-2,7-di-*tert*-butyl-9,9-dimethyl-9*H*-thioxanthene

**DOI:** 10.1107/S1600536812020624

**Published:** 2012-05-19

**Authors:** Omayra H. Rubio, Angel L. Fuentes de Arriba, Francisca Sanz, Francisco M. Muniz, Joaquín R. Morán

**Affiliations:** a Departamento de Química Orgánica, Universidad de Salamanca, Plaza de los Caídos, 37008 Salamanca, Spain; b Servicio Difracción de Rayos X, Universidad de Salamanca, Plaza de los Caídos, 37008 Salamanca, Spain; c Instituto de Cerámica y Vidrio, CSIC, Kelsen 5, 28049 Madrid, Spain

## Abstract

In the title compound, C_23_H_28_Br_2_S, the thioxanthene unit is twisted, showing a dihedral angle of 29.3 (5)° between the benzene rings. When projected along [001], the packing shows two types of channels. The crystal studied was a racemic twin.

## Related literature

For the preparation, see: Emslie *et al.* (2006 ▶). For the use of the title compound as a starting material in the preparation of rigid ligands for different transition metals, see: Emslie *et al.* (2008 ▶).
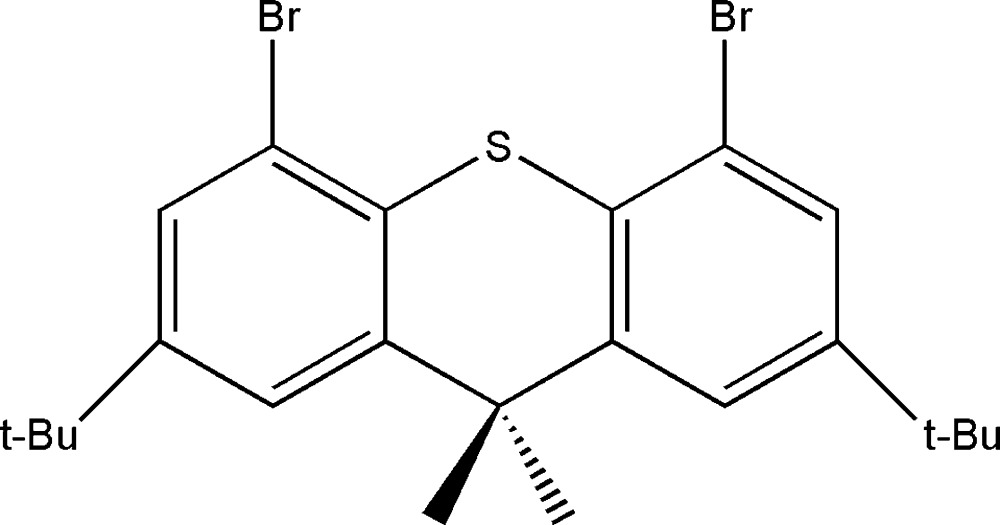


## Experimental

### 

#### Crystal data

C_23_H_28_Br_2_S
*M* *_r_* = 496.33
Tetragonal, 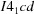

*a* = 21.8234 (2) Å
*c* = 18.8025 (5) Å
*V* = 8954.9 (3) Å^3^
*Z* = 16
Cu *K*α radiation
μ = 5.48 mm^−1^
*T* = 298 K
0.12 × 0.10 × 0.08 mm

#### Data collection

Bruker APEXII CCD area-detector diffractometer
Absorption correction: multi-scan (*SADABS*; Bruker, 2006 ▶) *T*_min_ = 0.544, *T*_max_ = 0.645
26972 measured reflections
3272 independent reflections
3038 reflections with *I* > 2σ(*I*)
*R*_int_ = 0.045

#### Refinement

*R*[*F* ^2^ > 2σ(*F* ^2^)] = 0.040
*wR*(*F* ^2^) = 0.114
*S* = 1.08
3272 reflections
243 parameters
1 restraint
H-atom parameters constrained
Δρ_max_ = 0.34 e Å^−3^
Δρ_min_ = −0.83 e Å^−3^
Absolute structure: Flack (1983 ▶), 2739 Friedel pairs
Flack parameter: 0.49 (3)

### 

Data collection: *APEX2* (Bruker 2006 ▶); cell refinement: *SAINT* (Bruker 2006 ▶); data reduction: *SAINT*; program(s) used to solve structure: *SHELXS97* (Sheldrick, 2008 ▶); program(s) used to refine structure: *SHELXL97* (Sheldrick, 2008 ▶); molecular graphics: *Mercury* (Macrae *et al.*, 2008) ▶; software used to prepare material for publication: *SHELXL97*.

## Supplementary Material

Crystal structure: contains datablock(s) global, I. DOI: 10.1107/S1600536812020624/ng5262sup1.cif

Structure factors: contains datablock(s) I. DOI: 10.1107/S1600536812020624/ng5262Isup2.hkl

Supplementary material file. DOI: 10.1107/S1600536812020624/ng5262Isup3.cml

Additional supplementary materials:  crystallographic information; 3D view; checkCIF report

## Figures and Tables

**Fig. 1.**
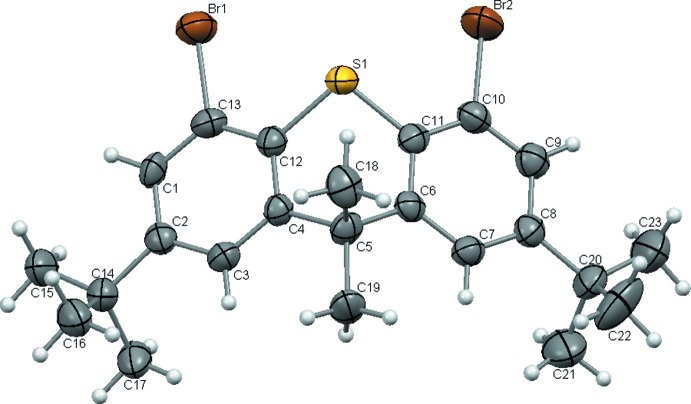
Molecular structure of C23H28Br2S. Displacement ellipsoids are drawn at the 50% probability level. Hydrogen atoms are shown as spheres of arbitrary radius.

**Fig. 2.**
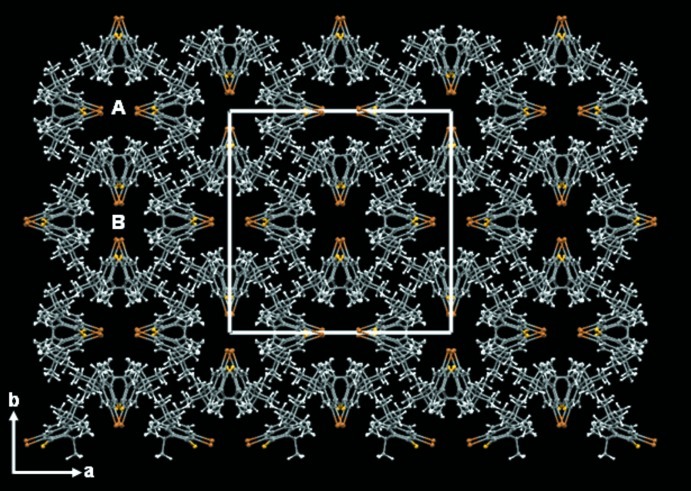
Crystal packing of C23H28Br2S view along c-axis, showing two kind of channels.

**Fig. 3.**
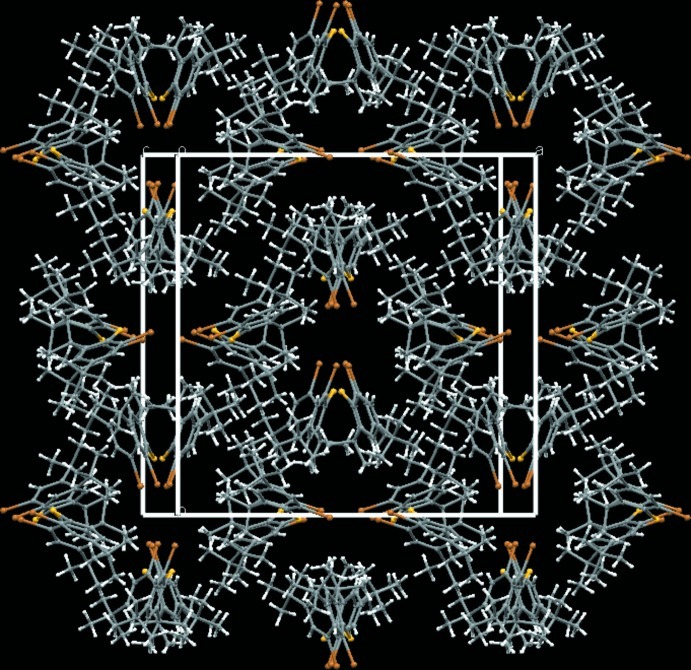
Crystal packing showed in Figure 2 moved along x axis.

## supplementary crystallographic information

### Comment

Thioxanthenes are very valuable building blocks for several purposes. Specifically, the compound described in this paper has been used as a starting material in the preparation of rigid ligands for different transitions metals as Ni, Pd, Fe, *etc* (Emslie *et al.*, 2008).

The crystal contains an unique molecule as the asymmetric unit. The molecule consists of a thioxanthene framework with a *tert*-butyl group at C2 and C8, two methyl groups at C5 and a bromine atom at C10 and C13 as susbtituents.The thioxanthene core is twisted with a torsion angle of 29.3 (5)° (C11—S1—C12—C4). All the bond lengths and angles are within the normal ranges. The S1—C11 and S1—C12 bond lengths are 1.751 (5) Å and 1.769 (5) Å, and the C11—S1—C12 angle are 99.5 (2)°. The bromine atoms are coplanar with the thioxanthene framework; the Br1—C13—C1—C2 and Br2—C10—C9—C8 torsion angles are 179.8 (2)° and -179.8 (9)°, respectively.

The molecules in the cell unit are orientated in opposite directions forming parallel sheets along the *a* and *b* axes, which intersect perpendicularly originating two types of channels A and B, as is shown in Fig. 2 and 3.

### Experimental

The title compound was obtained from thioxanthone according to a method described previously (Emslie *et al.*, 2006). Thioxanthone reacted with AlMe_3_ to gave 9,9-dimethylthioxanthene. This compound (0.75 g, 3.31 mmol) was mixed with 2-chloro-2-methylpropane (1.04 ml, 9.56 mmol) in chloroform (18 ml) at 273 K and aluminium trichloride (0.26 g, 1.95 mmol) was added in a Friedel-Crafts procedure. Reaction of this compound (0.57 g, 1.68 mmol) with bromine (0.34 ml, 6.64 mmol) in a mixture of glacial acetic acid (6.8 ml) and dichloromethane (3 ml) gave 2,7-di-*tert*-butyl-4,5-dibromo-9,9-dimethylthioxanthene. Crystals were obtained from a dichloromethane solution and their characterization was in agreement with the reported data.

### Refinement

The hydrogen atoms were positioned geometrically, with C—H distances constrained to 0.93 Å (aromatic CH) and 0.96 Å (methyl CH_3_) and refined in riding mode with *U*_iso_(H) = xUeq(C), where *x* =1.5 for methyl H atoms and *x* = 1.2 for all other atoms.

### Crystal data

**Table:**

C_23_H_28_Br_2_S	*D*_x_ = 1.473 Mg m^−^^3^
*M**_r_* = 496.33	Cu *K*α radiation, λ = 1.54178 Å
Tetragonal, *I*4_1_*c**d*	Cell parameters from 6340 reflections
Hall symbol: I 4bw -2c	θ = 4.1–65.0°
*a* = 21.8234 (2) Å	µ = 5.48 mm^−^^1^
*c* = 18.8025 (5) Å	*T* = 298 K
*V* = 8954.9 (3) Å^3^	Prism, brown
*Z* = 16	0.12 × 0.10 × 0.08 mm
*F*(000) = 4032	

### Data collection

**Table:**

Bruker APEXII CCD area-detector diffractometer	3272 independent reflections
Radiation source: fine-focus sealed tube	3038 reflections with *I* > 2σ(*I*)
Graphite monochromator	*R*_int_ = 0.045
phi and ω scans	θ_max_ = 67.1°, θ_min_ = 4.1°
Absorption correction: multi-scan (*SADABS*; Bruker, 2006)	*h* = −24→24
*T*_min_ = 0.544, *T*_max_ = 0.645	*k* = −25→24
26972 measured reflections	*l* = −21→19

### Refinement

**Table:**

Refinement on *F*^2^	Secondary atom site location: difference Fourier map
Least-squares matrix: full	Hydrogen site location: inferred from neighbouring sites
*R*[*F*^2^ > 2σ(*F*^2^)] = 0.040	H-atom parameters constrained
*wR*(*F*^2^) = 0.114	*w* = 1/[σ^2^(*F*_o_^2^) + (0.069*P*)^2^ + 8.551*P*] where *P* = (*F*_o_^2^ + 2*F*_c_^2^)/3
*S* = 1.08	(Δ/σ)_max_ < 0.001
3272 reflections	Δρ_max_ = 0.34 e Å^−^^3^
243 parameters	Δρ_min_ = −0.83 e Å^−^^3^
1 restraint	Absolute structure: Flack (1983), 2739 Friedel pairs
Primary atom site location: structure-invariant direct methods	Flack parameter: 0.49 (3)

### Special details

**Table:**

Geometry. All e.s.d.'s (except the e.s.d. in the dihedral angle between two l.s. planes) are estimated using the full covariance matrix. The cell e.s.d.'s are taken into account individually in the estimation of e.s.d.'s in distances, angles and torsion angles; correlations between e.s.d.'s in cell parameters are only used when they are defined by crystal symmetry. An approximate (isotropic) treatment of cell e.s.d.'s is used for estimating e.s.d.'s involving l.s. planes.
Refinement. Refinement of *F*^2^ against ALL reflections. The weighted *R*-factor *wR* and goodness of fit *S* are based on *F*^2^, conventional *R*-factors *R* are based on *F*, with *F* set to zero for negative *F*^2^. The threshold expression of *F*^2^ > σ(*F*^2^) is used only for calculating *R*-factors(gt) *etc*. and is not relevant to the choice of reflections for refinement. *R*-factors based on *F*^2^ are statistically about twice as large as those based on *F*, and *R*- factors based on ALL data will be even larger.

### Fractional atomic coordinates and isotropic or equivalent isotropic displacement parameters (Å^2^)

**Table:**

	*x*	*y*	*z*	*U*_iso_*/*U*_eq_	
Br1	0.00721 (3)	0.08500 (2)	0.15791 (4)	0.0637 (2)	
Br2	−0.01257 (4)	0.08030 (3)	0.44522 (4)	0.0759 (3)	
S1	0.01309 (5)	0.15662 (5)	0.30465 (9)	0.0469 (3)	
C1	−0.0512 (2)	0.1937 (2)	0.1083 (2)	0.0399 (10)	
H1	−0.0539	0.1723	0.0657	0.048*	
C2	−0.07413 (19)	0.2520 (2)	0.1132 (3)	0.0397 (10)	
C3	−0.0652 (2)	0.2836 (2)	0.1773 (3)	0.0396 (10)	
H3	−0.0796	0.3235	0.1808	0.048*	
C4	−0.03594 (19)	0.25826 (19)	0.2354 (3)	0.0351 (9)	
C5	−0.0220 (2)	0.29427 (18)	0.3048 (3)	0.0414 (9)	
C6	−0.0430 (2)	0.2563 (2)	0.3677 (2)	0.0377 (10)	
C7	−0.07826 (19)	0.2811 (2)	0.4233 (3)	0.0416 (10)	
H7	−0.0916	0.3214	0.4192	0.050*	
C8	−0.0943 (2)	0.2484 (2)	0.4845 (3)	0.0417 (10)	
C9	−0.0731 (2)	0.1878 (2)	0.4897 (3)	0.0463 (11)	
H9	−0.0815	0.1645	0.5299	0.056*	
C10	−0.0395 (2)	0.1630 (2)	0.4339 (3)	0.0442 (11)	
C11	−0.02527 (19)	0.1948 (2)	0.3734 (3)	0.0393 (10)	
C12	−0.0171 (2)	0.1968 (2)	0.2308 (3)	0.0384 (10)	
C13	−0.02398 (19)	0.1666 (2)	0.1671 (3)	0.0416 (10)	
C14	−0.1076 (2)	0.2842 (2)	0.0513 (3)	0.0436 (9)	
C15	−0.1193 (3)	0.2409 (3)	−0.0107 (3)	0.0638 (15)	
H15A	−0.1418	0.2621	−0.0471	0.096*	
H15B	−0.1427	0.2064	0.0055	0.096*	
H15C	−0.0809	0.2270	−0.0296	0.096*	
C16	−0.0707 (3)	0.3385 (3)	0.0251 (3)	0.0598 (15)	
H16A	−0.0629	0.3659	0.0640	0.090*	
H16B	−0.0933	0.3596	−0.0113	0.090*	
H16C	−0.0324	0.3244	0.0059	0.090*	
C17	−0.1701 (2)	0.3078 (3)	0.0782 (3)	0.0581 (14)	
H17A	−0.1639	0.3342	0.1184	0.087*	
H17B	−0.1951	0.2736	0.0921	0.087*	
H17C	−0.1902	0.3302	0.0410	0.087*	
C18	0.0481 (2)	0.3047 (2)	0.3083 (3)	0.0550 (12)	
H18A	0.0577	0.3303	0.3483	0.083*	
H18B	0.0616	0.3243	0.2654	0.083*	
H18C	0.0686	0.2660	0.3134	0.083*	
C19	−0.0520 (3)	0.35770 (19)	0.3027 (4)	0.0557 (12)	
H19A	−0.0956	0.3532	0.2975	0.084*	
H19B	−0.0361	0.3805	0.2632	0.084*	
H19C	−0.0433	0.3791	0.3461	0.084*	
C20	−0.1332 (2)	0.2772 (3)	0.5422 (3)	0.0521 (13)	
C21	−0.1930 (3)	0.3015 (4)	0.5106 (4)	0.080 (2)	
H21A	−0.2188	0.3164	0.5482	0.119*	
H21B	−0.2136	0.2691	0.4857	0.119*	
H21C	−0.1841	0.3343	0.4782	0.119*	
C22	−0.0982 (3)	0.3323 (4)	0.5735 (5)	0.093 (3)	
H22A	−0.0622	0.3180	0.5977	0.140*	
H22B	−0.1241	0.3537	0.6065	0.140*	
H22C	−0.0865	0.3596	0.5358	0.140*	
C23	−0.1493 (4)	0.2334 (4)	0.6019 (4)	0.091 (2)	
H23A	−0.1132	0.2244	0.6291	0.136*	
H23B	−0.1655	0.1962	0.5823	0.136*	
H23C	−0.1795	0.2520	0.6322	0.136*	

### Atomic displacement parameters (Å^2^)

**Table:**

	*U*^11^	*U*^22^	*U*^33^	*U*^12^	*U*^13^	*U*^23^
Br1	0.0755 (4)	0.0508 (3)	0.0647 (5)	0.0202 (2)	−0.0148 (4)	−0.0147 (3)
Br2	0.1107 (6)	0.0513 (3)	0.0655 (5)	0.0278 (3)	−0.0034 (5)	0.0074 (3)
S1	0.0510 (6)	0.0474 (5)	0.0423 (6)	0.0192 (4)	−0.0057 (7)	−0.0050 (6)
C1	0.045 (2)	0.046 (2)	0.029 (2)	−0.0015 (18)	−0.0009 (19)	−0.0120 (19)
C2	0.035 (2)	0.045 (2)	0.039 (3)	0.0011 (16)	0.0009 (19)	0.001 (2)
C3	0.044 (2)	0.036 (2)	0.039 (3)	0.0047 (17)	0.002 (2)	−0.0038 (18)
C4	0.036 (2)	0.036 (2)	0.033 (2)	−0.0014 (16)	−0.0028 (17)	−0.0045 (19)
C5	0.049 (2)	0.0350 (19)	0.040 (2)	−0.0015 (15)	0.000 (2)	−0.008 (2)
C6	0.040 (2)	0.038 (2)	0.035 (2)	0.0025 (16)	−0.0108 (19)	−0.0035 (18)
C7	0.044 (2)	0.038 (2)	0.043 (3)	0.0067 (16)	−0.005 (2)	−0.006 (2)
C8	0.039 (2)	0.049 (2)	0.037 (3)	0.0031 (18)	−0.0048 (18)	−0.003 (2)
C9	0.052 (3)	0.044 (2)	0.043 (3)	−0.0006 (19)	−0.007 (2)	−0.002 (2)
C10	0.050 (2)	0.038 (2)	0.045 (3)	0.0033 (18)	−0.009 (2)	0.002 (2)
C11	0.035 (2)	0.043 (2)	0.039 (3)	0.0047 (17)	−0.0084 (19)	−0.008 (2)
C12	0.037 (2)	0.043 (2)	0.035 (3)	0.0047 (17)	−0.0027 (19)	−0.005 (2)
C13	0.040 (2)	0.036 (2)	0.049 (3)	0.0064 (16)	0.002 (2)	−0.005 (2)
C14	0.048 (2)	0.048 (2)	0.035 (2)	0.0067 (18)	0.000 (3)	−0.001 (2)
C15	0.082 (4)	0.067 (3)	0.042 (3)	0.023 (3)	−0.015 (3)	−0.010 (3)
C16	0.063 (3)	0.069 (4)	0.048 (3)	0.010 (3)	0.005 (3)	0.013 (3)
C17	0.043 (3)	0.088 (4)	0.043 (3)	0.016 (3)	0.001 (2)	−0.001 (3)
C18	0.055 (3)	0.059 (3)	0.051 (3)	−0.012 (2)	−0.012 (3)	−0.004 (3)
C19	0.084 (3)	0.034 (2)	0.048 (3)	0.009 (2)	−0.001 (3)	−0.006 (3)
C20	0.049 (2)	0.063 (3)	0.044 (3)	0.011 (2)	0.009 (2)	−0.006 (2)
C21	0.061 (4)	0.099 (5)	0.078 (5)	0.027 (3)	0.011 (3)	0.007 (4)
C22	0.086 (5)	0.103 (5)	0.091 (6)	−0.004 (4)	0.021 (4)	−0.059 (5)
C23	0.103 (5)	0.102 (5)	0.066 (4)	0.035 (5)	0.030 (4)	0.010 (4)

### Geometric parameters (Å, º)

**Table:**

Br1—C13	1.915 (4)	C14—C17	1.543 (6)
Br2—C10	1.909 (4)	C15—H15A	0.9600
S1—C11	1.751 (5)	C15—H15B	0.9600
S1—C12	1.769 (5)	C15—H15C	0.9600
C1—C2	1.371 (7)	C16—H16A	0.9600
C1—C13	1.386 (7)	C16—H16B	0.9600
C1—H1	0.9300	C16—H16C	0.9600
C2—C3	1.402 (7)	C17—H17A	0.9600
C2—C14	1.544 (7)	C17—H17B	0.9600
C3—C4	1.381 (7)	C17—H17C	0.9600
C3—H3	0.9300	C18—H18A	0.9600
C4—C12	1.406 (7)	C18—H18B	0.9600
C4—C5	1.553 (7)	C18—H18C	0.9600
C5—C6	1.515 (7)	C19—H19A	0.9600
C5—C19	1.532 (6)	C19—H19B	0.9600
C5—C18	1.549 (6)	C19—H19C	0.9600
C6—C11	1.400 (7)	C20—C23	1.515 (10)
C6—C7	1.406 (7)	C20—C21	1.529 (8)
C7—C8	1.397 (7)	C20—C22	1.542 (9)
C7—H7	0.9300	C21—H21A	0.9600
C8—C9	1.405 (7)	C21—H21B	0.9600
C8—C20	1.514 (7)	C21—H21C	0.9600
C9—C10	1.389 (7)	C22—H22A	0.9600
C9—H9	0.9300	C22—H22B	0.9600
C10—C11	1.369 (7)	C22—H22C	0.9600
C12—C13	1.376 (7)	C23—H23A	0.9600
C14—C16	1.516 (8)	C23—H23B	0.9600
C14—C15	1.522 (8)	C23—H23C	0.9600
			
C11—S1—C12	99.5 (2)	C14—C15—H15C	109.5
C2—C1—C13	119.9 (4)	H15A—C15—H15C	109.5
C2—C1—H1	120.0	H15B—C15—H15C	109.5
C13—C1—H1	120.0	C14—C16—H16A	109.5
C1—C2—C3	117.6 (4)	C14—C16—H16B	109.5
C1—C2—C14	123.0 (4)	H16A—C16—H16B	109.5
C3—C2—C14	119.4 (4)	C14—C16—H16C	109.5
C4—C3—C2	123.3 (4)	H16A—C16—H16C	109.5
C4—C3—H3	118.4	H16B—C16—H16C	109.5
C2—C3—H3	118.4	C14—C17—H17A	109.5
C3—C4—C12	117.9 (4)	C14—C17—H17B	109.5
C3—C4—C5	123.6 (4)	H17A—C17—H17B	109.5
C12—C4—C5	118.5 (4)	C14—C17—H17C	109.5
C6—C5—C19	112.7 (4)	H17A—C17—H17C	109.5
C6—C5—C18	110.3 (4)	H17B—C17—H17C	109.5
C19—C5—C18	106.9 (4)	C5—C18—H18A	109.5
C6—C5—C4	108.6 (3)	C5—C18—H18B	109.5
C19—C5—C4	110.6 (4)	H18A—C18—H18B	109.5
C18—C5—C4	107.7 (4)	C5—C18—H18C	109.5
C11—C6—C7	117.5 (4)	H18A—C18—H18C	109.5
C11—C6—C5	120.0 (4)	H18B—C18—H18C	109.5
C7—C6—C5	122.4 (4)	C5—C19—H19A	109.5
C8—C7—C6	123.6 (4)	C5—C19—H19B	109.5
C8—C7—H7	118.2	H19A—C19—H19B	109.5
C6—C7—H7	118.2	C5—C19—H19C	109.5
C7—C8—C9	117.0 (4)	H19A—C19—H19C	109.5
C7—C8—C20	121.3 (4)	H19B—C19—H19C	109.5
C9—C8—C20	121.7 (5)	C8—C20—C23	113.6 (5)
C10—C9—C8	119.3 (5)	C8—C20—C21	110.1 (5)
C10—C9—H9	120.4	C23—C20—C21	108.0 (5)
C8—C9—H9	120.4	C8—C20—C22	108.6 (5)
C11—C10—C9	123.3 (4)	C23—C20—C22	108.9 (6)
C11—C10—Br2	120.2 (4)	C21—C20—C22	107.5 (6)
C9—C10—Br2	116.5 (4)	C20—C21—H21A	109.5
C10—C11—C6	119.2 (4)	C20—C21—H21B	109.5
C10—C11—S1	118.7 (4)	H21A—C21—H21B	109.5
C6—C11—S1	122.1 (4)	C20—C21—H21C	109.5
C13—C12—C4	118.6 (4)	H21A—C21—H21C	109.5
C13—C12—S1	119.2 (3)	H21B—C21—H21C	109.5
C4—C12—S1	122.2 (4)	C20—C22—H22A	109.5
C12—C13—C1	122.5 (4)	C20—C22—H22B	109.5
C12—C13—Br1	119.0 (3)	H22A—C22—H22B	109.5
C1—C13—Br1	118.5 (4)	C20—C22—H22C	109.5
C16—C14—C15	109.0 (5)	H22A—C22—H22C	109.5
C16—C14—C17	108.4 (4)	H22B—C22—H22C	109.5
C15—C14—C17	108.1 (4)	C20—C23—H23A	109.5
C16—C14—C2	110.4 (4)	C20—C23—H23B	109.5
C15—C14—C2	112.0 (4)	H23A—C23—H23B	109.5
C17—C14—C2	108.8 (4)	C20—C23—H23C	109.5
C14—C15—H15A	109.5	H23A—C23—H23C	109.5
C14—C15—H15B	109.5	H23B—C23—H23C	109.5
H15A—C15—H15B	109.5		
